# PKCδ Regulates Force Signaling during VEGF/CXCL4 Induced Dissociation of Endothelial Tubes

**DOI:** 10.1371/journal.pone.0093968

**Published:** 2014-04-03

**Authors:** Joshua Jamison, James H-C. Wang, Alan Wells

**Affiliations:** 1 Department of Pathology, McGowan Institute for Regenerative Medicine, University of Pittsburgh, Pittsburgh, Pennsylvania, United States of America; 2 Department of Orthopedic Surgery, McGowan Institute for Regenerative Medicine, University of Pittsburgh, Pittsburgh, Pennsylvania, United States of America; University of Birmingham, United Kingdom

## Abstract

Wound healing requires the vasculature to re-establish itself from the severed ends; endothelial cells within capillaries must detach from neighboring cells before they can migrate into the nascent wound bed to initiate angiogenesis. The dissociation of these endothelial capillaries is driven partially by platelets' release of growth factors and cytokines, particularly the chemokine CXCL4/platelet factor-4 (PF4) that increases cell-cell de-adherence. As this retraction is partly mediated by increased transcellular contractility, the protein kinase c-δ/myosin light chain-2 (PKCδ/MLC-2) signaling axis becomes a candidate mechanism to drive endothelial dissociation. We hypothesize that PKCδ activation induces contractility through MLC-2 to promote dissociation of endothelial cords after exposure to platelet-released CXCL4 and VEGF. To investigate this mechanism of contractility, endothelial cells were allowed to form cords following CXCL4 addition to perpetuate cord dissociation. In this study, CXCL4-induced dissociation was reduced by a VEGFR inhibitor (sunitinib malate) and/or PKCδ inhibition. During combined CXCL4+VEGF treatment, increased contractility mediated by MLC-2 that is dependent on PKCδ regulation. As cellular force is transmitted to focal adhesions, zyxin, a focal adhesion protein that is mechano-responsive, was upregulated after PKCδ inhibition. This study suggests that growth factor regulation of PKCδ may be involved in CXCL4-mediated dissociation of endothelial cords.

## Introduction

Wounds prevent skin from acting as a barrier between the body and external harsh environment, a vital function that must be quickly re-established during wound repair. This repair requires a healthy vascular system for both tissue generation and subsequent maintenance. Blood vessels are compromised during wounding which require angiogenesis from the tips of severed vessels for regeneration. This is driven by pro-angiogenic growth factors released first by platelets and then by macrophages in the wound. Endothelial cells that are involved in angiogenesis require initial signals to ‘dedifferentiate’ and separate from the existing severed vessels prior to subsequent inductive signals to migrate into the wound bed. These early wound response signals to initiate angiogenesis are mediated through platelets during clotting including the chemokine CXCL4 and growth factors VEGF, PDGF, HB-EGF, and TGF [1].

Many of the intracellular signaling pathways that drive fibroblasts and endothelial cells to migrate are known. Downstream of growth factor receptor activation, PLCγ1 signaling triggers PKCδ to regulate cell motility via increasing transcellular contractility in fibroblasts and endothelial cells [2]–[8]. Growth factor and matrikine signaling through the epidermal growth factor receptor (EGFR) initiates motility via phosphorylation and activation of PLCγ1 at the membrane [3]. Activated PLCγ1 then catalyzes the hydrolysis of PIP_2_ (primarily at the leading edge of the membrane) and generates diacylglycerol (DAG) and IP3 [9], [10]. Increased levels of DAG at the leading edge [11] synergizes the effect of PKCδ to the membrane [12]. DAG subsequently stabilizes the activation of PKCδ through direct binding of its N-terminal C1 domain [13]–[15] Furthermore, PKCδ localization behind the leading edge allows it to propel the cell body towards the extended lamellipodium and also mediate isometric force associated with motility [16]. It has also been previously demonstrated that downstream of PKCδ signaling, an intermediate kinase, specifically myosin light chain kinase (MLCK), relays signaling to MLC through direct phosphorylation [8]. Further supporting EGFR induced regulation of contractility, it was demonstrated that reduced activation of PLCγ1 delayed subsequent activation of PKCδ and downstream MLC-2 [8]. These data have shown that EGFR triggers contractile responses efficiently and quickly through the PLCγ1/PKCδ pathway.

These findings led us to ask whether such a pathway could also account for the initial retraction of severed vessels prior to angiogenic sprouting. Increased PKCδ activation has also been shown to increase vascular permeability [17]–[19], consistent with cell-cell de-adherence. Inhibition of PKCδ results in downregulation of stress fibers and focal adhesions in endothelial cells [20]. In addition, steady-state activation of stress fiber tension by PKCδ has been found to strengthen the endothelial cell barrier [21], [22]. All of these data implicate biphasic PKCδ regulation in maintaining vascular permeability. Furthermore, MLCK that is downstream of PKCδ is involved in endothelial retraction through MLC-2 phosphorylation. These signals initiating endothelial retraction may be essential for the initial phases of endothelial cell separation from damaged vessels prior to angiogenic sprouting. However, the mechanism in which PKCδ mediates endothelial cell retraction has not been determined.

Therefore, we hypothesized that PKCδ regulates the tension of endothelial cords by promoting cell retraction during neo-angiogenesis through VEGFR signaling in addition to CXCL4 mediated retraction.

## Results

### CXCL4+VEGF-induced dissociation is VEGFR-dependent

**Figure 1:**
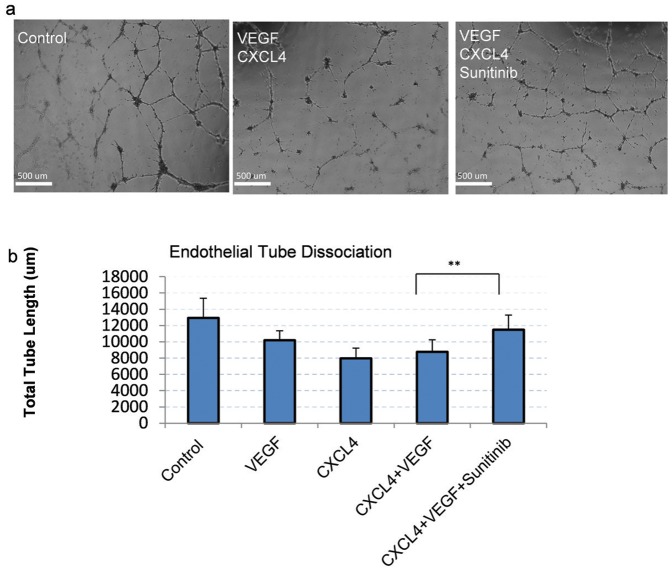
VEGFR inhibition decreases CXCL4-induced cord dissociation. a) Representative phase contrast images of HMEC cells treated with indicated treatments are shown. Images were taken of live cords after 24 hours. Disruption in webbed patterning of cells indicates increased dissociation. b) Images described in (**Fig 1a**) were quantified as described in methods using Metamorph analysis software (N at least 4; mean ± SD, ** P<0.05), Shown are representative of 3 experiments.

To investigate endothelial CXCL4-mediated dissociation, we used human microvascular endothelial cells (HMEC-1) plated on Matrigel. Cell cords were given 24 hours to form and then subsequently induced to dissociate with CXCL4 and VEGF, two factors released by platelets during the initial stages of hemostasis. Inhibition of VEGFR/PDGFR signaling using sunitinib (2.5 uM) inhibited dissociation, as demonstrated by longer cord length (**Fig. 1**). In addition, CXCL4 had a more pronounced effect than VEGF on mediating tube dissociation (**Fig 1**).

### VEGFR/CXCL4-induced dissociation is partially PKCδ-dependent

**Figure 2:**
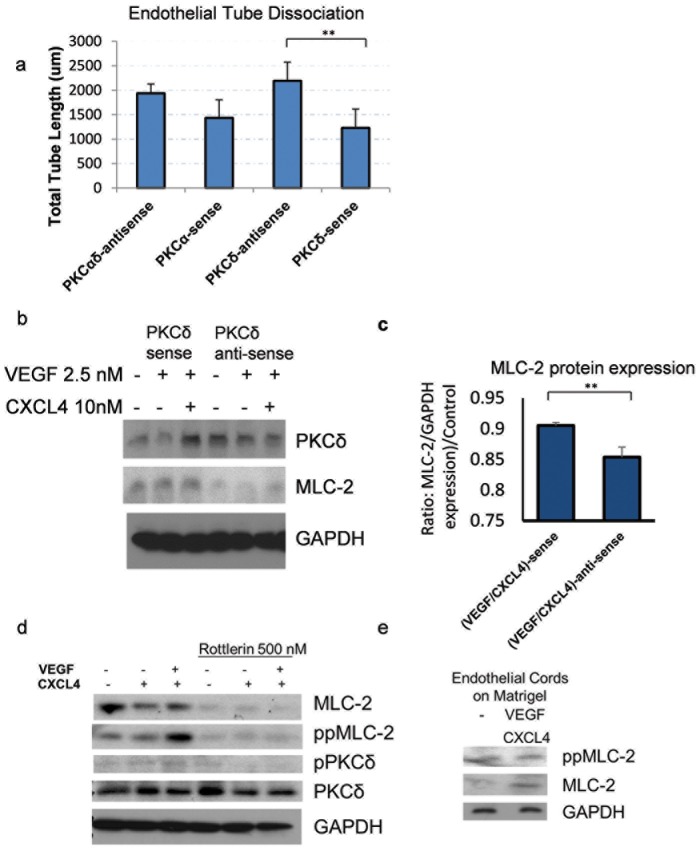
PKCδ inhibition decreases CXCL4-induced cord dissociation. a) Cords that were induced to dissociate were quantified using Metamorph as in (Fig. 1b). HMEC cells were allowed to form cords. Following 24 hours, 20 uM of antisense/sense oligonucleotides were added to cords as described in methods and allowed to dissociate with inhibition of PKCδ for 24 hours. Cords and cord length were measured (N at least 4; mean ± SD ** P<0.05); blots shown are representative of 3 experiments. b) Immunoblot analysis of HMEC monolayer lysates during a 24 hour addition of CXCL4 (10 nM) and VEGF (2.5 nM) in the presence of antisense inhibition. GAPDH was used as a loading control. c) MLC expression was analyzed from **(** **Fig 2b** **)** with either PKCδ antisense or sense in response to CXCL4 (10 nM) and VEGF (2.5 nM) (N = 3; mean ± SD ** P<0.05). d) Primary adult dermal human microvascular endothelial cells were treated with indicated CXCL4 (10 nM) and VEGF (2.5 nM) in the presence and absence of PKCδ inhibitor (Rottlerin 500 nM) for 24 hours. Cell lysates were analyzed using immunoblot analysis. GAPDH was used as a loading control, as the control representative of the two blots shown. e) HMEC-1 cells were plated on Matrigel to form cords in a 6-well plate. After cords were formed, cells were treated with CXCL4 (10 nM) and VEGF (2.5 nM) for 24 hours. Western blot analysis was performed on lysates of both endothelial cords and Matrigel. GAPDH was used as a loading control.

Tube dissociation involves the separation of cell-cell contacts, we therefore investigated whether transcellular contractility involves PKCδ, a key signaling nexus in contractility and tension [7], [8]. Dissociation was blunted by downregulation of PKCδ. Formed cords were stimulated to dissociate with CXCL4 and VEGF in the presence or absence of antisense towards PKCδ. Endothelial cord length reduction by CXCL4/VEGF was partially rescued by antisense against PKCδ (**Fig. 2a**). PKCδ downregulation attenuated cord dissociation to a significantly lesser degree. In addition, the antisense decreased PKCδ and MLC-2 levels that are downstream of PKCδ regulation (**Fig. 2b** **,** **Fig 2c**). In addition, adult primary endothelial cells were investigated, as we found that MLC-2 was also down regulated through rottlerin inhibition, a PKCδ inhibitor (**Fig. 2d**). In response to VEGF/CXCL4, ppMLC-2 levels in adult primary endothelial cells were increased, in contrast to CXCL4 only treatment (**Fig. 2d**). To further investigate endothelial cords themselves for increased contractility, HMEC-1 endothelial cord lysates were analyzed in which showed increased MLC-2 levels in response to VEGF/CXCL4 **(** **Fig. 2e** **)**. These data indicate that PKCδ affects MLC-2 expression in a VEGF-dependent manner, which in turn correlates with its regulation of stress fibers.

### Dynamics of endothelial cords/capillaries tension require PKCδ-dependent motility of cords and individual cells

**Figure 3:**
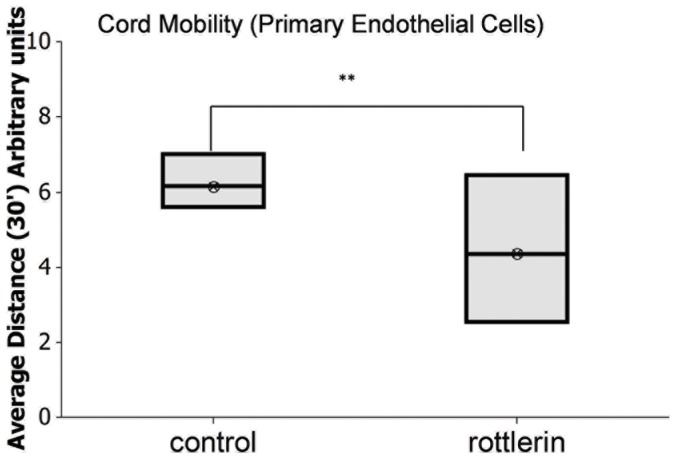
Fiogure 3. PKCδ inhibition limits the mobilization/tension on the cords. Quantification of cord motion in supplemental movies 1 and 2. Live cell imaging of cords as they move were quantified with metamorph analysis (Sup. Movie 1/2), as each cord excluding branches were tracked and quantified as average distance displacement for 30′ (N = 14 cords measured; mean ± SD **P<0.01).

Cell motility is critical for vessel formation and vessel regression [23]. To further investigate the role of PKCδ activation during VEGF/CXCL4 mediated dissociation, adult primary endothelial cords were induced to dissociate in the presence of rottlerin (500 nM). Cord motion that is a part of the process of vessel maturation was observed in both groups, however PKCδ inhibition through rottlerin reduced cord mobility (**Fig 3**, Sup. Movie 1,2). According to these data, dissociation was mediated by the motility of the cells and PKCδ-dependent regulation of cytoskeleton/isometric contractions.

We further investigated cord dissociation/mobility and found increased mobility and contractions in cords that were treated with CXCL4/VEGF (**Movies S3, S4**). In addition, increased force was exerted onto the Matrigel by the endothelial cells, as shown by the deformation of the substratum noted in live cell imaging via phase contrast microscopy. Individual endothelial cells at their junctions were compressed and spheroid as this morphology appeared to enable the necessary movements for increased mobility for subsequent cord collapse (**Movie S4**).

### Activation of PKCδ is increased during CXCL4/VEGF-induced dissociation of endothelial cords

**Figure 4:**
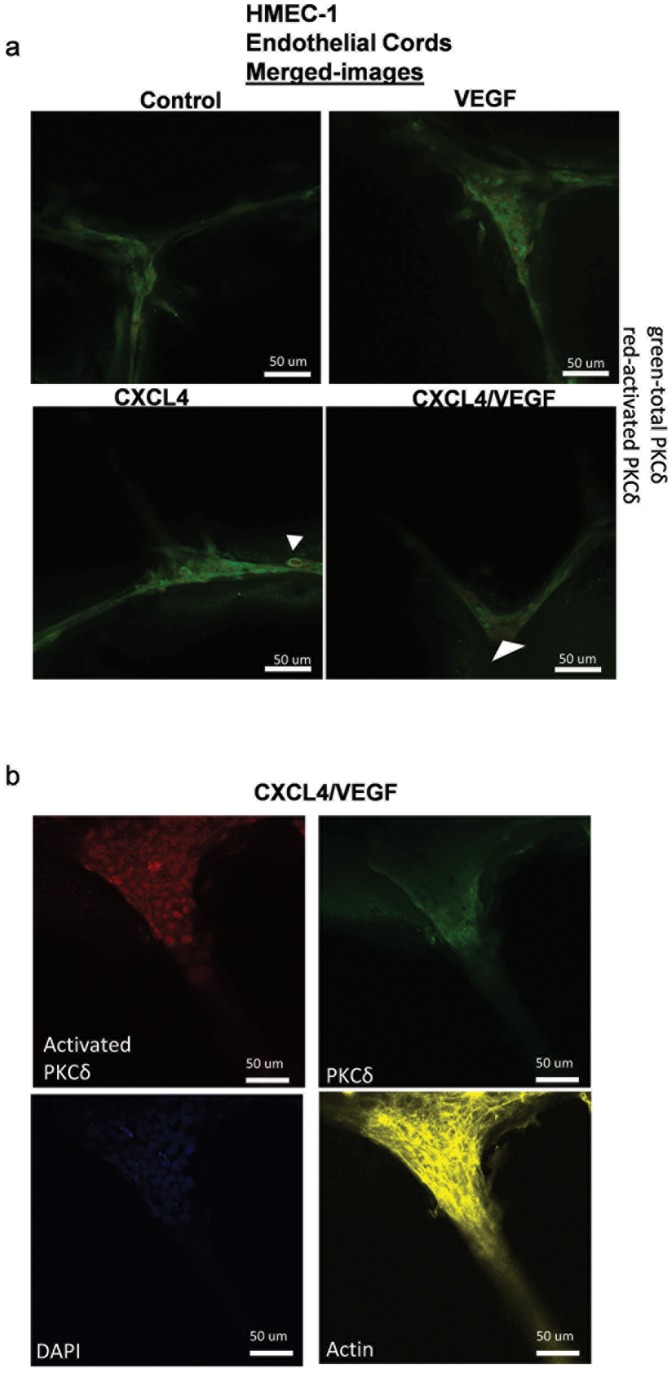
PKCδ is activated during cord dissociation. a/b) Immunostaining of HMEC endothelial cells formed into cords onto Matrigel treated with indicated treatments for 24 hours after cords are formed. a) Merged representative images of activated PKCδ (red) and PKCδ (green) for indicated treatments. b) Representative image of CXCL4 100 nM/VEGF 2.5 nM treated cord that was immunostained with PKCδ and phospho-PKCδ indicated in green and orange separate images. Cells were also stained for nuclei with DAPI staining represented as blue and actin with phalloidin staining (Alexa-633) represented in pseudo-color yellow. Representative image of a larger cord indicates increased effects of force translated in the ratio of activated PKCδ to PKCδ. Arrows of increased phosphorylated PKCδ expressing cells indicates positioning of possibly active cells at branch points of cords.

To further investigate whether PKCδ was activated during dissociation, levels of PKCδ and activated (or phosphorylated) PKCδ were measured in endothelial cords. In CXCL4+VEGF-induced cells, this ratio was increased at junctions with increased phosphorylated PKCδ at junction edges and decreased PKCδ in the inner part of the junction (**Fig. 4a**). In addition, in CXCL4 only- and CXCL4+VEGF-mediated dissociation, endothelial cells with a spheroid morphology and increased levels of activated PKCδ were also observed. Although PKCδ has been shown in previous literature to mediate apoptosis [24], [25]}, it seems that these cells may actually alter their mesenchymal phenotype to incorporate into the cords and to actively mediate tension at junctions (**Fig 4b**, **Movie S4).** This would be consistent with our finding that PKCδ directing cell contractility [7]. These data imply that activation of PKCδ is associated with force generation and possibly force mechanics in endothelial cord dissociation.

### Mechanotransduction of VEGF/CXCL4 induced cord dissociation is PKCδ-dependent

**Figure 5:**
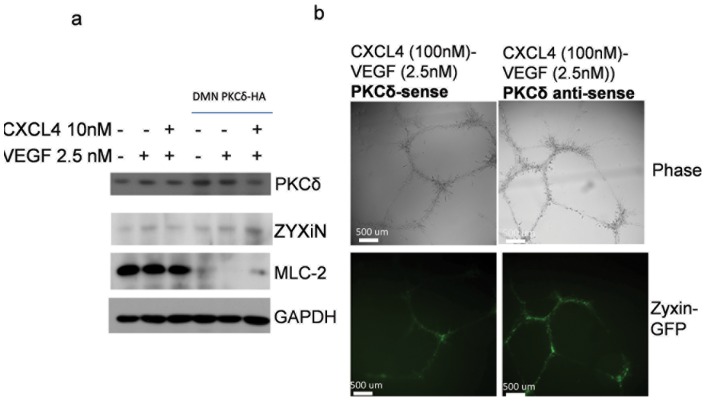
The tension-sensor zyxin is elevated in the absence of PKCδ. a) Immunoblot of HMEC monolayer transfected with Dominant negative PKCδ DNA vector (4 ug/lipofectamine[10 ul]) prior to treatment with CXCL4/VEGF in quiescence media for 24 hours. b) Live cell fluorescent images of Zyxin-expressing HMEC cells that have formed cords. After cords were formed, cells were incubated for 5 days with 20 uM PKCδ-sense or PKCδ-anti-sense in VEGF 2.5 nM/CXCL4 100 nM. Shown are representative of 3 experiments.

Cord dissociation involves multiple intercellular forces which coordinate the subsequent collapse of capillaries. To confirm that force impacts the endothelial cords molecularly, focal adhesions/junctions were examined as cellular locations affected by CXCL4/VEGF induced dissociation. As a focal adhesion protein, the zyxin protein has been previously found regulated in response to vascular stretch. In this study, zyxin was used to investigate mechanosensory input into the cords as they dissociate. The investigation of zyxin showed that its expression is linked to PKCδ activity, as inhibition of PKCδ caused increased zyxin expression in 2D monolayer (Fig 5a). Furthermore, downregulation of PKCδ with antisense oligonucleotides in formed endothelial cords led to increased zyxin expression (Fig 5b). These data suggest focal adhesions are dynamically regulated when force is induced. Furthermore, PKCδ that is inhibited by a dominant negative construct causes focal adhesions to stabilize, thus contributing to inhibition of cord dissociation.

These data suggest that force signaling through VEGFR is applied during cord dissociation through motility signaling via PKCδ. Signaling through VEGFR causes PKCδ to elicit both static and dissociative regulation of force for endothelial capillary stability and dissociation, respectively.

## Discussion

The CXCL4 receptor, CXCR3, primarily regulates endothelial capillary dissociation through calcium regulation. When calcium channels are stretch-activated, u-calpain is activated that results in cleavage of focal adhesions leading to cell de-adherence and subsequent anoikis/apoptosis [26]. In this study, we investigated VEGFR signaling to PKCδ as a non-direct molecular mediator of CXCL4-induced cord/capillary dissociation. Based on the results of this study, PKCδ that is activated downstream of VEGFR\tyrosine kinase signaling plays a role in endothelial cell dissociation (**Fig. 1**). Attenuation of cord dissociation through inhibition of VEGFR and PKCδ indicates that, for full dissociation of cords on Matrigel, VEGFR signaling through PKCδ is required (**Fig. 1a**, **Fig. 1b**). VEGFR regulation also mediates increased regulation of PKCδ, as seen in our data when PKCδ levels were upregulated (**Fig. 1d**). PKCδ regulation of contractility through MLC-2 was observed in data where PKCδ antisense and PKCα antisense caused MLC-2 downregulation after CXCL4-VEGF treatment (**Fig. 1d**). However, the effect of PKCα antisense was lower, and may affect PKCδ activity indirectly through an unknown mechanism. We further inhibited the dissociation response using the pharmacological agent rottlerin and by causing the cell cords to become static and non-motile (**Movie S1, S2, Fig. 3**). Since motility is essential for cord formation, our data also highlight the importance of cord integrity (**Movies S3**), which translates to cord retraction and dissociation (**Movie S4**).

PKCδ activation was also investigated during cord dissociation. The ratio of activated PKCδ to PKCδ was increased at junctions in CXCL4-VEGF-treated cells (**Fig. 4**). Moreover, some cells that were activated with PKCδ were circular and were centrally located within junctions (**Fig 4**). It is interesting to consider whether these cells are functionally important in integrating cord force as the cords dissociate or move. Further experiments are needed to fully test this hypothesis. If further investigation supports this idea, it would offer new insight into how PKCδ regulates motility and force in microvasculature.

We further showed that cord motility and dissociation are directly linked to PKCδ regulation. PKCδ seems to contribute to dissociation more by affecting the plasticity of active movement than by disrupting intracellular junctions mediated by calpains (**Fig 1**). In this investigation, we show that PKCδ regulates dissociation through VEGFR to PLCγ1 signaling. However, it remains unknown whether force signaling is directing the dissociation, or recursively responding to force regulation. Both situations require PKCδ, but in a different context. These results also demonstrate that motility mechanisms seen in 2D migration play an important role in multi-cellular tissue. Increased calcium signaling perpetuates increased contraction and de-adhesion, while VEGFR signaling is also mediating and directing contractions through PKCδ signaling.

After dissociation has occurred, stabilization of PKCδ may still mediate further contraction of cords in the right context [21], [22], [27]. As cords shift to a more stabilized and quiescent cellular state, signaling from VEGFR and other receptors with intrinsic tyrosine kinase activity continues in order to further activate downstream mediators of PKCδ. These events fine-tune the development of a normal vascular structure and help stabilize autocrine signaling. Alternately, angiogenic driven VEGF stimulation induces upregulation of PKCδ that would result in destabilization of the vasculature, with the separated endothelial cells now being responsive to motile signaling. This is combined with extracellular signaling of other molecular mediators and regulation by the stroma, allowing for remodeling of the wound bed. Lastly, pericytes also regulate the microvasculature with force regulation [28], [29]. Studies investigating how pericytes regulate cord stability/dissociation would further elucidate how force signaling is modulated *in vivo*. Such studies represent the next step in discerning the function of cord mobility in vascular regeneration.

## Methods

### HMEC-1 cell culture

HMEC-1 (human dermal microvascular endothelial cells) were obtained from CDC, Atalanta, Georgia, originally described by Ades et al [30]. Endothelial cells were grown in MCDB 131 (Gibco) media with 10 mM L-glutamine supplemented with 10% fetal bovine serum. For stable expression of the zyxin protein, HMEC-1 cells were transfected with 4 ug of GFP-Zyxin plasmid (obtained from Origene) with 10 uL of lipofectamine in 6 well dish with subconfluent cells. Stable selection of Zyxin focal adhesion markers in HMEC-1 cells was established with neomycin selection (350 ng/mL) as cells were passaged.

### Adult-Dermal Human Microvascular Endothelial Cell culture

Primary human endothelial cells were obtained from (Life Technologies) and were grown in EGM-2MV Media, (Lonza). Cells were grown 5 passages prior to incubating cells in quiescent media for 24 hour before experiment.

### Matrigel tube formation assay

Cells were grown to 50% confluency prior to seeding onto Matrigel. Growth Factor Reduced (GFR) Matrigel (BD Biosciences) was seeded onto a u-chamber slide at 10 ul per well or 120 uL per well on a 24-well plate and incubated for 30 minutes at 37°C for polymerization. Cells were then re-plated onto Matrigel at 75,000 cells per well (on the 24-well plate) or 15,000 cells per well (on the u-chamber slide, Ibidi) for 24 hours. Cords were grown overnight in quiescent media 0.5% FBS. Afterwards, endothelial cords were allowed to dissociate with VEGF-165 Recombinant growth factor and Recominbnat CXCL4 (PeproTech), which were added at 2.5 nM and 100 nM respectively after 24 hours (or as indicated by the time points given in figure/movie). Some experiments were quantified for cord length by adding 2.5 uM Cell Tracker Green for better quantification.

### Antisense inhibition

DNA oligonucleotides sequences targeting PKCδ and PKCα were obtained from the literature [31], [32] and ordered from IDT; phospho-thioate bonds were added to the end nucleotides. Oligonucleotides sense and anti-sense of human PKCδ are GTGGCATGATGGAGCCTTTT and 5′TTTTCCGAGGTAGTACCGTG-3′, respectively. Oligonucleotide sense and antisense PKCα are 5′-CGGGCAACGACTCCACGGCG-3′ and ‘5-CGC CGT GGA GTC GTT GCC CG-3’, respectively. DNA oligonucleotides at 20 uM were added after HMEC cords were formed in quiescence media, and additional antisense was added to the cells via lipofectamine (10 uL) for 0.5 ug of DNA in 500 uL of OPTI medium per sample in 6 well plate. DNA/Lipofectamine was added to quiescent media overnight prior to growth factor/cytokine treatment.

### Live cell imaging

HMEC-1/Primary endothelial cells were grown and plated in a 6 well glass bottom plate on Matrigel at 75,000 cells per well and incubated in quiescent media overnight. Endothelial cords that formed were treated with indicated treatments and immediately added to live cell chamber with 5% O_2_, 5% CO_2_, or 90% N_2_ at 37°C. Primary endothelial cells (Fig. 3, Movie S1 Movie S2) were imaged for 7 hours and HMEC-1 cells were imaged for 1 hour and 20 minutes at 5 minute intervals (Movie S3 and Movie S4). Images were taken of endothelial cords on top of Matrigel with a Nikon Eclipse Ti live cell imaging microscope at 10× and 20× objective respectively as size bars are indicated in live cell imaging movies.

### Immunostaining/confocal microscopy

HMEC-1 cells cultured and plated onto 15 well u-chamber slides (Ibidi) as previously described. After selected treatments, cells were fixed in 2% formaldehyde in PBS for 10 minutes. Cells were then permeabilized with 0.1% Triton-X-100 in PBS (wash buffer) for 10 minutes. Afterwards, cells were washed for 30 minutes (3× washes) prior to incubation with antibodies. Antibodies were diluted (1∶50) for anti-phospho-PKC-theta/delta(S643/S676)(Cell Signaling) polyclonal rabbit and (1∶100) anti-PKCδ (polyclonal anti-mouse) in wash buffer with 30 mg of bovine serum albumin. Cells were immunostained overnight at 4°C. Cells were then washed for 15 minutes (3×) and incubated with secondary antibody at (1∶100) (Alexa-488-antimouse and Alexa-594-antirabbit- LifeTechnologies) in wash buffer/30 mg of BSA/5% goat serum, as secondary antibodies were goat. Antibodies were incubated onto cells for 1 hour at room temperature before cells were washed (3×). Alexafluor-633 phalloidin (1∶40) in PBS and DAPI (1∶10000) was added to cells. Afterwards, cells were desiccated for 60′ at RT to decrease matrigel depth (z) above 180 um in micro-chamber slide. PBS was added to cells for 30 minutes prior to imaging and were imaged on an Olympus Fluoview FV1000 confocal IX81 microscope.

### Metamorph analysis

Total cord length was measured using Metamorph analysis software. Mininimum cord length was set at 1 um or 1 pixel and max cord width was set to 30 um. The track object function in metamorph was used to track cord motion.

### Statistical Analysis

One-way ANOVA was performed (Fig. 1) with significance being determined at p<0.05. Two sample Student's T test was performed for all other experiments with significance being determined at p<0.05.

## Supporting Information

Movie S1 **Representative movie of adult primary dermal human microvascular endothelial cords in quiescent media.** After cords were formed rottlerin (500 nM) was added to group (Movie S2) immediately before imaging. Cell tracker orange was added at 2.5 uM concentration for better observation of groups immediately before imaging. Endothelial cords were imaged 7 hours for 30 minutes per frame, as movie represents merged images of red fluorescence and phase. Scale bar of 50 um is indicated on lower right hand corner; images were taken at 10× objective lens.
(AVI)

Movie S2 **Representative movie of adult primary dermal human microvascular endothelial cords in quiescent media.** After cords were formed rottlerin (500 nM) was added to group (Movie S2) immediately before imaging. Cell tracker orange was added at 2.5 uM concentration for better observation of groups immediately before imaging. Endothelial cords were imaged 7 hours for 30 minutes per frame, as movie represents merged images of red fluorescence and phase. Scale bar of 50 um is indicated on lower right hand corner; images were taken at 10× objective lens.
(AVI)

Movie S3 **Representative movies of HMEC-1 endothelial cords were taken in either quiescent media or with CXCL4 (100 nM)/VEGF (2.5 nM) added immediately before imaging (Movie S4).** Phase contrast images were taken for a duration of 1 hour 20 minutes at 5 minutes per frame. Scale bar of 50 um is indicated.
(AVI)

Movie S4 **Representative movies of HMEC-1 endothelial cords were taken in either quiescent media or with CXCL4 (100 nM)/VEGF (2.5 nM) added immediately before imaging (Movie S4).** Phase contrast images were taken for a duration of 1 hour 20 minutes at 5 minutes per frame. Scale bar of 50 um is indicated on lower right hand corner; images were taken at 20× objective lens.
(AVI)

## References

[1] Li J, Zhang YP, Kirsner RS (2003) Angiogenesis in wound repair: angiogenic growth factors and the extracellular matrix. Microsc Res Tech 60: 107–114. 12500267 10.1002/jemt.10249

[2] Chen P, Gupta K, Wells A (1994) Cell movement elicited by epidermal growth factor receptor requires kinase and autophosphorylation but is separable from mitogenesis. J Cell Biol 124: 547–555. 8106552 10.1083/jcb.124.4.547 PMC2119923

[3] Chen P, Xie H, Sekar MC, Gupta K, Wells A (1994) Epidermal growth factor receptor-mediated cell motility: phospholipase C activity is required, but mitogen-activated protein kinase activity is not sufficient for induced cell movement. J Cell Biol 127: 847–857. 7962064 10.1083/jcb.127.3.847 PMC2120228

[4] Shizukuda Y, Tang S, Yokota R, Ware JA (1999) Vascular endothelial growth factor-induced endothelial cell migration and proliferation depend on a nitric oxide-mediated decrease in protein kinase Cdelta activity. Circ Res 85: 247–256. 10436167 10.1161/01.res.85.3.247

[5] Yamamura S, Nelson PR, Kent KC (1996) Role of protein kinase C in attachment, spreading, and migration of human endothelial cells. J Surg Res 63: 349–354. 8661224 10.1006/jsre.1996.0274

[6] Joyce NC, Meklir B (1992) Protein kinase C activation during corneal endothelial wound repair. Invest Ophthalmol Vis Sci 33: 1958–1973. 1582801

[7] Jamison J, Lauffenburger D, Wang JC, Wells A (2013) PKCdelta Localization at the Membrane Increases Matrix Traction Force Dependent on PLCgamma1/EGFR Signaling. PLoS One 8: e77434. 24155954 10.1371/journal.pone.0077434 PMC3796482

[8] Iwabu A, Smith K, Allen FD, Lauffenburger DA, Wells A (2004) Epidermal growth factor induces fibroblast contractility and motility via a protein kinase C delta-dependent pathway. J Biol Chem 279: 14551–14560. 14747473 10.1074/jbc.M311981200

[9] Wells A, Ware MF, Allen FD, Lauffenburger DA (1999) Shaping up for shipping out: PLCgamma signaling of morphology changes in EGF-stimulated fibroblast migration. Cell Motil Cytoskeleton 44: 227–233. 10602252 10.1002/(SICI)1097-0169(199912)44:4<227::AID-CM1>3.0.CO;2-B

[10] Insall RH, Weiner OD (2001) PIP3, PIP2, and cell movement—similar messages, different meanings? Dev Cell 1: 743–747. 11740936 10.1016/s1534-5807(01)00086-7 PMC2819114

[11] Shao H, Chou J, Baty CJ, Burke NA, Watkins SC, et al (2006) Spatial localization of m-calpain to the plasma membrane by phosphoinositide biphosphate binding during epidermal growth factor receptor-mediated activation. Mol Cell Biol 26: 5481–5496. 16809781 10.1128/MCB.02243-05 PMC1592705

[12] Ron D, Kazanietz MG (1999) New insights into the regulation of protein kinase C and novel phorbol ester receptors. FASEB J 13: 1658–1676. 10506570

[13] Stahelin RV, Digman MA, Medkova M, Ananthanarayanan B, Melowic HR, et al (2005) Diacylglycerol-induced membrane targeting and activation of protein kinase Cepsilon: mechanistic differences between protein kinases C-delta and C-epsilon. J Biol Chem 280: 19784–19793. 15769752 10.1074/jbc.M411285200

[14] Seki T, Matsubayashi H, Amano T, Shirai Y, Saito N, et al (2005) Phosphorylation of PKC activation loop plays an important role in receptor-mediated translocation of PKC. Genes Cells 10: 225–239. 15743412 10.1111/j.1365-2443.2005.00830.x

[15] Kikkawa U, Matsuzaki H, Yamamoto T (2002) Protein kinase C delta (PKC delta): activation mechanisms and functions. J Biochem 132: 831–839. 12473183 10.1093/oxfordjournals.jbchem.a003294

[16] Andujar MB, Melin M, Guerret S, Grimaud JA (1992) Cell migration influences collagen gel contraction. J Submicrosc Cytol Pathol 24: 145–154. 1600506

[17] Lum H, Malik AB (1996) Mechanisms of increased endothelial permeability. Can J Physiol Pharmacol 74: 787–800. 10.1139/y96-081 8946065

[18] Lum H, Malik AB (1994) Regulation of vascular endothelial barrier function. Am J Physiol 267: L223–241. 7943249 10.1152/ajplung.1994.267.3.L223

[19] Lynch JJ, Ferro TJ, Blumenstock FA, Brockenauer AM, Malik AB (1990) Increased endothelial albumin permeability mediated by protein kinase C activation. J Clin Invest 85: 1991–1998. 2347922 10.1172/JCI114663 PMC296668

[20] Tinsley JH, Teasdale NR, Yuan SY (2004) Involvement of PKCdelta and PKD in pulmonary microvascular endothelial cell hyperpermeability. Am J Physiol Cell Physiol 286: C105–111. 13679307 10.1152/ajpcell.00340.2003

[21] Fordjour AK, Harrington EO (2009) PKCdelta influences p190 phosphorylation and activity: events independent of PKCdelta-mediated regulation of endothelial cell stress fiber and focal adhesion formation and barrier function. Biochim Biophys Acta 1790: 1179–1190. 19632305 10.1016/j.bbagen.2009.07.012 PMC2759355

[22] Harrington EO, Shannon CJ, Morin N, Rowlett H, Murphy C, et al (2005) PKCdelta regulates endothelial basal barrier function through modulation of RhoA GTPase activity. Exp Cell Res 308: 407–421. 15935342 10.1016/j.yexcr.2005.05.005

[23] Stokes CL, Lauffenburger DA, Williams SK (1991) Migration of individual microvessel endothelial cells: stochastic model and parameter measurement. J Cell Sci 99 (Pt 2): 419–430. 1885678 10.1242/jcs.99.2.419

[24] Geraldes P, Hiraoka-Yamamoto J, Matsumoto M, Clermont A, Leitges M, et al (2009) Activation of PKC-delta and SHP-1 by hyperglycemia causes vascular cell apoptosis and diabetic retinopathy. Nat Med 15: 1298–1306. 19881493 10.1038/nm.2052 PMC3290906

[25] Shizukuda Y, Helisch A, Yokota R, Ware JA (1999) Downregulation of protein kinase cdelta activity enhances endothelial cell adaptation to hypoxia. Circulation 100: 1909–1916. 10545436 10.1161/01.cir.100.18.1909

[26] Bodnar RJ, Yates CC, Wells A (2006) IP-10 blocks vascular endothelial growth factor-induced endothelial cell motility and tube formation via inhibition of calpain. Circ Res 98: 617–625. 16484616 10.1161/01.RES.0000209968.66606.10 PMC3826264

[27] Grinnell KL, Harrington EO (2012) Interplay between FAK, PKCdelta, and p190RhoGAP in the regulation of endothelial barrier function. Microvasc Res 83: 12–21. 21549132 10.1016/j.mvr.2011.04.005 PMC3175025

[28] Murphy DD, Wagner RC (1994) Differential contractile response of cultured microvascular pericytes to vasoactive agents. Microcirculation 1: 121–128. 8790583 10.3109/10739689409148267

[29] Lee S, Zeiger A, Maloney JM, Kotecki M, Van Vliet KJ, et al (2010) Pericyte actomyosin-mediated contraction at the cell-material interface can modulate the microvascular niche. J Phys Condens Matter 22: 194115. 21386441 10.1088/0953-8984/22/19/194115

[30] Ades EW, Candal FJ, Swerlick RA, George VG, Summers S, et al (1991) HMEC-1: establishment of an immortalized human microvascular endothelial cell line. J Invest Dermatol 99: 683–690. 10.1111/1523-1747.ep12613748 1361507

[31] Li Q, Subbulakshmi V, Fields AP, Murray NR, Cathcart MK (1999) Protein kinase calpha regulates human monocyte O-2 production and low density lipoprotein lipid oxidation. J Biol Chem 274: 3764–3771. 9920929 10.1074/jbc.274.6.3764

[32] Woo CH, Lim JH, Kim JH (2005) VCAM-1 upregulation via PKCdelta-p38 kinase-linked cascade mediates the TNF-alpha-induced leukocyte adhesion and emigration in the lung airway epithelium. Am J Physiol Lung Cell Mol Physiol 288: L307–316. 15489375 10.1152/ajplung.00105.2004

